# *Cis*-regulatory landscapes in the evolution and development of the mammalian skull

**DOI:** 10.1098/rstb.2022.0079

**Published:** 2023-07-03

**Authors:** Marketa Kaucka

**Affiliations:** Max Planck Institute for Evolutionary Biology, Plön 24306, Germany

**Keywords:** morphological evolution, enhancer, skull development, craniofacial variability, differential gene expression

## Abstract

Extensive morphological variation found in mammals reflects the wide spectrum of their ecological adaptations. The highest morphological diversity is present in the craniofacial region, where geometry is mainly dictated by the bony skull. Mammalian craniofacial development represents complex multistep processes governed by numerous conserved genes that require precise spatio-temporal control. A central question in contemporary evolutionary biology is how a defined set of conserved genes can orchestrate formation of fundamentally different structures, and therefore how morphological variability arises. In principle, differential gene expression patterns during development are the source of morphological variation. With the emergence of multicellular organisms, precise regulation of gene expression in time and space is attributed to *cis*-regulatory elements. These elements contribute to higher-order chromatin structure and together with *trans*-acting factors control transcriptional landscapes that underlie intricate morphogenetic processes. Consequently, divergence in *cis*-regulation is believed to rewire existing gene regulatory networks and form the core of morphological evolution. This review outlines the fundamental principles of the genetic code and genomic regulation interplay during development. Recent work that deepened our comprehension of *cis*-regulatory element origin, divergence and function is presented here to illustrate the state-of-the-art research that uncovered the principles of morphological novelty.

This article is part of the theme issue ‘The mammalian skull: development, structure and function’.

## 1. Introduction

A series of new features and transformations separate modern mammals from extant reptiles and their common ancestors. These include endothermy, the double circulatory system and four-chambered heart, mammary glands, hair, the presence of a diaphragm and a spectrum of morphological novelties within the craniofacial compartment [1] (for a popular summary of fossil findings, see [2]). In the head, two important structures, the nervous system and the skull, underwent several coordinated changes. For instance, encephalization was linked to cranial vault expansion and separation of middle ear bones from the jaw allowed the development of superior hearing. Increased complexity of the turbinals and expansion of the respiratory and olfactory neuroepithelium occurred and enabled improvement of sense of smell [3–7]. Among other changes in the craniofacial skeletal system are heterodonty, the appearance of flexible joints in the hyoid apparatus and enlargement of the dentary bone [8].

Similar to other vertebrates, the mammalian skull provides structural support and protection and is characterized by complex shape [7]. Hosting the breathing and feeding apparatus, together with structures fulfilling sensory functions such as vision, olfaction and hearing, the formation of proper skull morphology is key for survival. Skull shape reflects adaptation of a given species to a certain ecological niche, feeding behaviour and overall lifestyle [9,10]. The general shape of the skull and face is known to have high heritability and is extraordinarily polygenic [11].

Mammalian craniofacial development is a lengthy process coordinated by a large number of conserved genes [12–14]. Head formation uses cells from all germ layers, and they undergo extensive proliferation and specification, resulting in the patterning and integration of a spectrum of tissues and organs [13,15]. The individual skeletal elements underlying head geometry emerge independently and asynchronously to fuse later and form the skull [16,17]. Importantly, the general blueprint of skull shape is established early during embryogenesis [17,18]. The core molecular components orchestrating craniofacial formation are genes from conserved families such as WNT, FGF, Hedgehog and BMP. Robust yet precise regulation of their expression is fundamental for the coordination of the head and skull formation and dynamic gene expression patterns are at the core of development. This raises the question of how molecular and developmental processes are calibrated to achieve such impressive shape variability as is found in mammals. It is now recognized that inter- and intra-species morphological divergence is driven principally through quantitative and spatio-temporal changes in gene expression.

Gene expression programmes are controlled by *cis*-regulatory elements and *trans*-acting factors that jointly form gene regulatory networks (GRNs). These regulatory assemblies determine when, where and to what extent a particular gene will be transcribed throughout development [19]. *Cis*-regulatory elements comprise promoters, enhancers, silencers and boundary elements. *Trans*-regulation relies on proteins such as transcription factors (TFs) and cofactors, and non-coding transcripts with regulatory function such as enhancer RNAs or long-non-coding RNAs. Recent research has highlighted that the *cis*-regulatory and *trans*-acting systems have differential evolutionary rates, rendering *cis*-regulators a prime target for evolution [20,21]. Moreover, increasing evidence of non-coding loci association with congenital disorders and trait-associated variants has boosted interest in understanding *cis*-regulation and its combinatorial complexity.

Advances in whole-genome sequencing, comparative and functional genomics and computational strategies allowed the identification of genome-wide regulatory landscapes and confidently linked enhancer structural and functional divergence along phylogeny with the emergence of novel traits and morphological variance [22]. Large numbers of candidate *cis*-regulatory elements have been discovered; however, reliable functional validations are still ongoing. While extensive research has been carried out in the last decades, many puzzle pieces are still missing to provide us with a holistic understanding of *cis*-regulatory evolution and impact on complex developmental processes.

In this review, I summarize emerging principles of enhancer evolution and their function in orchestrating spatio-temporal gene expression in mammals, to highlight how genomic calibration of conserved genes alters morphogenetic processes. To understand how morphological variation arises during embryogenesis, we must integrate large-scale functional and comparative genomics with detailed knowledge of molecular and cellular processes and tissue patterning.

## 2. Enhancers

Since their discovery more than 40 years ago, enhancers have been known as potent modulators of gene expression [23–25] (for a personal perspective on enhancer discovery, see [26]). Together with promoters and boundary elements, enhancers represent a fundamental mechanism regulating transcription. These *cis*-regulatory elements together with *trans*-acting TFs and cofactors contribute to dynamic higher-order chromatin structure and regulate or facilitate developmental stage-specific or tissue-specific gene expression programmes [27].

Enhancers lie within the non-coding intergenic and intronic regions, although a small set of enhancers also reside within exons [28–30]. Enhancers can be located at various distances from a target gene promoter, up to 1000 kb away, both up- and downstream [31,32]. Enhancers control transcription over long genomic distances, irrespective of their position and orientation relative to the target gene promoter. Enhancers are typically short (100–1000 bp) sequences containing dense clusters of multiple diverse transcription factor-binding sites (TFBSs) [33]. Therefore, one *cis*-regulatory element can enhance the transcription of several genes. However, the number of enhancers greatly exceeds the number of protein-coding genes, thus, one gene is frequently regulated by several regulatory elements [34]. This allows utilization of a limited set of genes; each being regulated by multiple enhancers with different spatio-temporal activities. At the same time, the existence of enhancer redundancy renders them a prime target for evolution without risk of severe effects (lethality) [35,36]. In such cases, individual enhancers mostly control low expression level of the target gene and their joint effect results in a high expression level of their target gene. Such clusters of enhancers may act as super enhancers that control the expression level of essential genes, for instance, genes controlling cell fate identity. This extraordinary combinatorial complexity of enhancer(s)–promoter interactions makes the precise annotation of dynamically changing transcriptional regulatory networks challenging.

**Figure 1.**
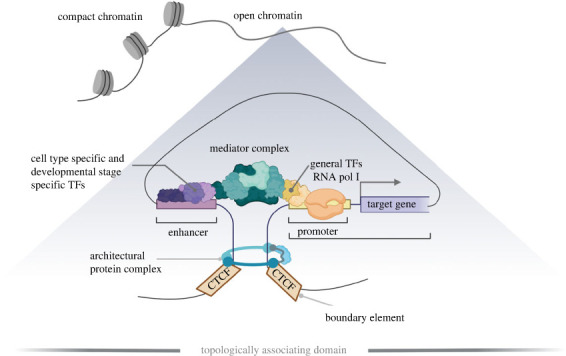
Illustration of chromatin structure and the transcription-regulating mechanism. The boundary elements with architectural protein complexes contribute to DNA looping and formation of the topologically associating domain (TAD). The TAD represents a fundamental unit of three-dimensional genome organization and allows physical interaction of the DNA sequences within (brings enhancers and genes into proximity). The specific combination of transcription factor-binding motifs within the enhancer enables docking of cell type specific or developmental stage specific transcription factors. This is followed by the recruitment of gene-specific or general transcription factors and a mediator complex, and the initiation of transcription.

**Figure 2.**
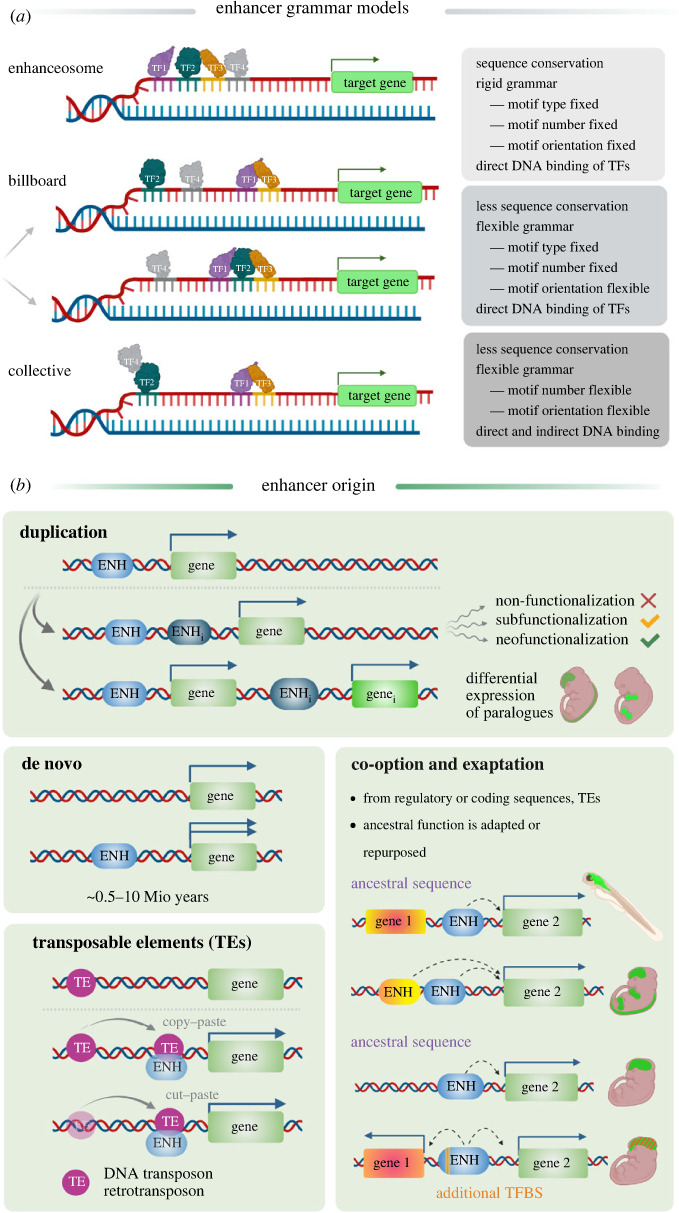
Schematic overview of (*a*) the enhancer grammar models and (*b*) modes of enhancer origin.

**Figure 3.**
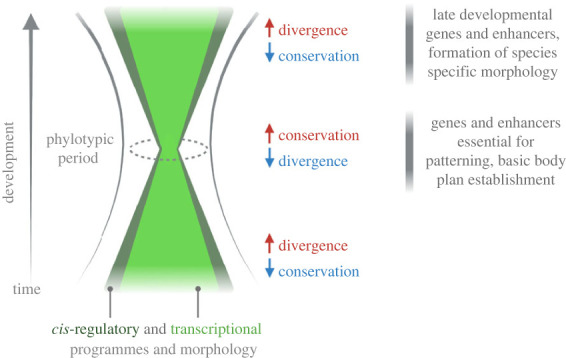
Conservation and divergence of both the *cis*-regulatory and transcriptional landscapes resemble the hour-glass model of embryonic evolution. The hour-glass model was originally proposed based on the observations that in the early and late embryonic stages, there is increased morphological divergence, while at the mid-embryonic stage—referred to as phylotypic period—the embryos within each animal phylum are morphologically similar. The conservation and divergence of genes, expression programmes and *cis*-elements also follow this pattern during embryonic development [119–121]. Deep conservation and low divergence of the mid-embryonic period is considered a source of the basic body plan formation, thus the active enhancers at this defined period are mostly associated with essential developmental genes that control patterning. Increasing *cis*- and molecular divergence in the later stages govern species-specific developmental programmes and acquisition of distinct morphologies.

Enhancers can physically or indirectly recruit or interact with transcription factors and cofactors and together with chromatin architectural proteins (e.g. Cohesin, CTCF) contribute to higher-order chromatin structure (figure 1) [27,37]. Two main models of enhancer architecture have been originally proposed (figure 2*a*). In the ‘enhanceosome’ model [29,38–40], the invariable number, type, order, orientation and spacing of TF motifs is required for enhancer activity [41]. The function of such enhancers requires deep sequence conservation as a mutation in one of the TFBSs will affect the functionality of the whole regulatory element [33,42]. Deeply conserved enhancers are mostly located in the proximity of genes coding for TFs essential to development (figure 3) and their mutation is often linked to human congenital syndromes [31,43,44]. In the second model of enhancer architecture, known as the ‘billboard model’ [29,40,45], the specific combination of TF motifs, irrespective of their order and orientation, defines enhancer activity and robustness. However, comparative whole-genome investigations of enhancer conservation across species, their evolution [46] and synthetic enhancer reporter studies [47] suggest that enhancers fall into both categories as well as in a spectrum between these two models, referred to as a TF-collective model of enhancer architecture. In the intermediate ‘collective model’, a specific set of TFs is needed for enhancer activation; however, the TFs can be recruited both directly by their respective TFBSs (DNA–protein) as well as indirectly (protein–protein).

In all enhancer architecture models, the binding of multiple TFs is required for enhancer activation (figure 1). Cooperative TF binding within the TFBS cluster of an enhancer enables the energetic barrier to be overcome resulting from an intrinsic affinity of the regulatory region to histone octamers, increasing DNA accessibility and transcription [33,48]. In principle, a higher number of TFBS in a regulatory element increases DNA accessibility and correlates with stronger expression of a target gene [49,50]. The TFs recruited by enhancers are often both lineage-specific and sequence-specific factors, thus integrating intrinsic and extrinsic developmental signalling cues and interpreting them in a precise context-specific manner [51].

Similar to promotors, active enhancers are nucleosome-free and can recruit the mediator and pre-initiation complex in addition to TFs (figure 1). Enhancers can be thus bidirectionally transcribed, although enhancer RNA levels are low and mostly rapidly degraded. Recently, it has been suggested that enhancer RNA contributes to transcriptional regulation [52,53], nevertheless, this hypothesis requires further support. However, there is a correlation between enhancer RNA presence and the overall enhancer activity [54–56].

## 3. Enhancer origin and evolution of their structural and functional variation

Enhancers heavily outnumber promoters and are known to have a higher mutation rate than protein-coding regions. This renders enhancers a substrate for evolution and structural divergence, and in turn, the diversification of multicellular organisms [21,57]. Early metazoans—multicellular organisms with complex cell type diversity—possessed enhancer-like regulatory elements and most metazoan chromosomes possess self-associating topological domains [58]. Metazoan enhancer sequences have been shown to activate genes in the vertebrates, highlighting the evolutionary conservation of their function regardless of structural conservation [59,60]. Nevertheless, deeply conserved enhancers are a minority in the mammalian genome [61]. Comparative and functional large-scale genomic studies identified enhancer emergence and loss to be common along phylogeny and suggested several mechanisms of enhancer evolution. Apart from metazoans, enhancers or enhancer-like regulatory elements have been identified in bacteria and plants, highlighting their ancient function in gene regulation and speciation [62,63].

*Cis*-regulatory elements can arise in multiple ways (figure 2*b*). In one scenario, enhancers emerge through co-option or exaptation from pre-existing regulatory or coding sequences [61,64]. This means that the ancestral sequence function has been adapted and repurposed to fulfil another role [61,64]. The second recognized mode of enhancer evolution is based on transposable elements (TEs). These are repetitive DNA elements originally described as selfish genetic elements, able to mobilize within the genome and cause genomic instability, act as another driving force in enhancer and gene regulation evolution [65–67]. TEs are of two classes, based on their structural properties and insertion mechanism that can be simply described as: copy-and-paste or cut-and-paste [68]. TEs form a significant proportion of mammalian genomes (up to 45% in human) and are enriched in enhancer regulatory regions [69–73]. Enhancer birth, driven by TE-insertion or TE-excision can either produce new enhancer activity or the mobilized sequence already contains regulatory function and enhances expression of a new target gene when inserted elsewhere. Mobile elements, both DNA transposons and retrotransposons, are thought to have contributed to evolutionary transitions and are believed to underlie the mammalian radiation [73–75]. The third mode of enhancer evolution is based on duplications of genomic regions, whole chromosomes or even whole genomes. The duplicated region may be lost (non-functionalization) or partially lost (sub-functionalization), or may gain new functional features (neofunctionalization) [76]. One copy is redundant and can tolerate mutations without causing adverse effects. If the duplicated sequence was fulfilling a regulatory function before, it may change to gain a new regulatory function. In case the whole enhancer-gene sequence was duplicated, the original and new regulatory elements may evolve to drive differential expression of the paralogues, as seen in zebrafish [77]. De novo enhancer evolution from a sequence that was not transcribed nor had regulatory function, is predicted to be an additional mechanism of enhancer evolution [78,79]. Nevertheless, *in silico* modelling of complex *cis*-regulatory element evolution has estimated that the time needed for de novo enhancer origin may vary greatly and range between 0.5 and 10 million years [80].

Originally, it was proposed that in vertebrates, enhancers were structurally (in terms of their sequence) and functionally conserved. Nevertheless, variable sequence divergence has been reported in functionally conserved elements [81,82]. Functionally conserved enhancers seem to have preferentially conserved specific TF motifs, though [83]. A recent study comparing the *cis*- and *trans*-regulatory landscapes of adult liver samples across 20 mammalian species revealed that enhancers evolve significantly faster than promoters and this differential evolution rate is a fundamental property of mammalian genomes [61]. Additionally, Villar and colleagues indicated that sequence constraint or TFBS density are poor predictors of enhancer conservation, rendering comparative sequence-based approaches for such investigations unsuitable. Most active enhancers identified in this study were positioned proximally to promotors. Interestingly, half of all enhancers in each species seemed to be recently evolved, which renders exaptation a widespread phenomenon, at least across placentals and redeployment of ancestral DNA a dominant mechanism to generate active enhancers across mammals [61]. While the composition and architecture of TFBS within enhancers is undoubtedly decisive for their activation properties, it has been suggested that regions flanking TFBS clusters additionally influence the binding of TFs that are sensitive to the local DNA shape [84,85].

## 4. Enhancers and the modulation of developmental programmes

Mechanisms controlling the plasticity of mammalian skull development contribute to the speciation and radiation of species. Skull formation is a multistep process with a series of cell and tissue specifications, driven by distinct sets of genes, and most craniofacial morphological variation is introduced after the phylotypic stage (figure 3). Morphogens that control proliferation, cell specification and tissue patterning have a prominent role in the process of skull formation and shaping [15,86–88]. Differential morphogen expression patterns are found among vertebrates and are regarded as a prominent source of morphological variability. Morphogens are mostly conserved genes, and thus their species-specific expression patterns have evolved through divergence of *cis*-regulation [22].

To dissect the impact of enhancers on complex developmental processes, it is essential to be aware of the order and interplay of individual developmental events. This is particularly challenging in head and skull development as this is a lengthy and intricate process. From the specification and migration of the neural crest cells, separation of placodal fields, polarized division of ectomesenchyme, induction of mesenchymal condensations, chondrocranium growth, until the onset of endochondral and intramembranous ossification, continuously increasing numbers of cell types interact to coordinate formation of multiple structures. Individual developmental steps follow each other, and so alteration at any part of embryonic space and at any point of embryonic time can trigger a sequence of follow-up changes. Alterations of various cellular processes (migration, polarity, proliferation) with tissue-level effects on patterning and integration will have diverse effects on morphogenesis. Therefore, knowledge of molecular orchestration and cellular communication is key to link the role of the *cis*-regulatory landscape to developmental processes.

Nowadays, it is acknowledged that the major source of morphological variability is heterochrony, the change of developmental timing, or heterotopy, alterations in spatio-temporal patterning of gene expression. As a consequence of such changes, many aspects of morphogenesis can be modulated, such as anisotropic proliferation rates, spatial distribution of proliferative zones and regions, qualitative and quantitative changes in cell specification, and alterations of tissue-specific cell polarity.

Enhancers, comprising a cluster of specific TFBSs, recruit a specific combination of TFs and can also dock them in a defined stepwise sequence, thus encoding precise information on cell type- or developmental stage-specific transcriptional signature. This allows the enhancer to respond to current developmental cues and the specification history of the cell. Enhancers are able to integrate multiple signals to control the spatio-temporal expression of their target genes in this way. Essential developmental genes tend to be associated with complex regulatory landscapes comprising many context-dependent enhancers [29]. While coding region mutations affect gene function in all cell types in which that gene is expressed, mutations in regulatory sequences usually have a more developmental stage and cell type-specific impact and lead to tissue-restricted phenotypic consequences. This led researchers interested in the pathogenesis of non-syndromic congenital craniofacial disorders to search for the underlying causes in non-coding regions. Examples are cleft palate or craniosynostosis, frequent congenital abnormalities in humans. In this case, the term non-syndromic refers to specific craniofacial abnormality with no further phenotypic manifestation in other organs or tissues. A specific example is the genetic basis of Pierre Robin syndrome that manifests as underdevelopment of the lower jaw and sometimes a cleft palate. Various extent deletions and inversions were found in a distant non-coding region known as the PRS sequence (Pierre Robin Sequence), far upstream of *SOX9*. Broad expression of *SOX9* throughout development (e.g. in the cranial neural crest cells (CNCCs) or chondrocytes), and its essential function in craniofacial formation, served as a paradigm for *in vitro* and *in vivo* testing of enhancer spatio-temporal effect. The authors showed that a cluster of enhancers within the PRS controlled *Sox9* expression in a narrow developmental window; during early facial formation and specifically in neural crest cells [32]. This is a great example of the defined effect of enhancers on a specific cell type or developmental stage.

## 5. Enhancers in inter- and intra-species craniofacial diversity

The availability of numerous mammalian genomes has fuelled comparative research of *cis*-regulatory evolution [89–91]. However, such investigations have mostly relied on computational approaches to identify conserved regulatory non-coding elements that underwent prominent structural change or were deleted [92]. Large-scale functional genomics enabled identification of *cis*-regulatory and transcriptional landscapes genome-wide, nevertheless, most studies employed conventional models of mammalian development (i.e. *Mus musculus*), whole postnatal organs or tissues for cross-species comparisons, or *in vitro* models [20,91,93,94]. While such approaches are very informative, efforts to identify and quantify driving forces behind the evolution of mammalian *cis*-regulatory landscapes have been hampered by the lack of high-resolution (i.e. on a single-cell level) regulatory maps from relevant tissues and developmental stages, that could be directly compared between species. Understanding the impact of a species-specific regulatory network on developmental and molecular dynamics during complex trait formation will be essential. For now, exactly how differential 4D gene expression patterns are translated into head and skull formation and shaping remains largely unexplored.

In recent years, an increasing amount of research has aimed to connect *cis*-regulatory evolution with the emergence of specific traits (both physiological or pathological). Enhancer fine-tuning of gene expression profiles is acknowledged as a key mechanism calibrating underlying developmental processes and controlling species-specific and individual-specific phenotypic traits [21]. In this section, I highlight recent pioneering work related to mammalian skull formation. Because craniofacial formation is an intricate multistep process employing dozens of cell types, comprehensive understanding of *cis*-regulatory evolution in mammalian craniofacial morphology is still rather in its beginnings.

A pioneering study by Attanasio *et al.* [95] analysed regulatory landscapes specifically in the facial tissues of the E11.5 mouse embryo. At this developmental stage, the major tissue patterning of all craniofacial prominences (medio-nasal, lateral nasal, maxillary and mandibular) is ongoing and most organogenesis in the head has been initiated. The authors identified nearly 4400 craniofacial distant-acting enhancers with both broad and tightly confined patterns in subregions of developing embryonic structures. Interestingly, the majority of candidate enhancers showed evolutionary conservation and the vast majority had orthologous sequences found in the human genome. The authors reported enrichment of enhancers in the proximity of genes associated with known craniofacial phenotypes in mouse mutant models and human patients. Later, a study analysing craniofacial shape variation within an outbred mouse population identified genetic loci associated with skull shape variation, with a high overlap of QTL regions with regulatory elements identified by Attanasio and co-workers [95,96]. These studies indicate that causal variants related to specific skull shape may be preferentially located in regulatory regions of the candidate genes.

**Figure 4.**
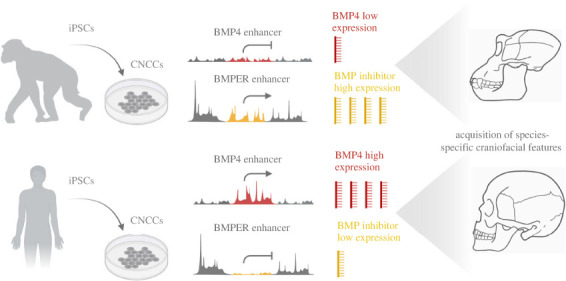
Simplified graphical representation of the study by Prescott *et al.* [93]. Induced pluripotent stem cells from human and chimpanzee were used for *in vitro* differentiation into cranial neural crest cells (CNCCs). Comparative epigenomics revealed divergence in *cis*-regulation between these closely related species. Numerous candidate enhancers were found in the proximity of craniofacial genes. Known morphogens (such as BMP4) and their regulators (BMPER; BMP inhibitor) are differentially regulated on the enhancer level and contribute to the formation of species-specific features.

An elegant study by Prescott and colleagues used induced pluripotent stem cells (iPSCs) derived from human and chimpanzees, our nearest living evolutionary relative. *In vitro* differentiation of iPSCs into CNCCs enabled comparative epigenomics to understand the logic of *cis*-regulatory evolution in closely related species (for simplified graphical representation, figure 4) [97]. CNCCs are multipotent cells specified within the borders of the folding anterior neural plate. Neural crest cells evolved in vertebrates and can give rise to a wide spectrum of derivatives (for a comprehensive overview, see [98]). The facial compartment is built in the majority from CNCCs, and thus comparing the regulatory and transcriptional landscape in this cell population is a suitable model to understand the effect of *cis*-regulatory divergence on facial shape formation and evolution. The authors found that genes implicated in facial development and morphology, such as *BMP4*, were associated with human-biased enhancers and expressed at higher levels. BMP4 is a well-known morphogen influencing facial morphology in birds and fish [86,99]. It is, therefore, not surprising that chimpanzee-associated enhancers were instead promoting higher expression of the BMP4-inhibitor *BMPER*. Previously, it has been reported that CNCC-specific overexpression of *Bmp4* in CNCCs in a mouse model dramatically altered skull shape and the changes resembled features typically different between human and chimps (with the mutant mouse model acquiring more human-specific features) [87]. Prescott and colleagues identified numerous enhancers in the proximity of genes known to regulate neural crest cell biology, and in turn, craniofacial morphology (e.g. *PAX3* and *PAX7*). Such genes were found strongly associated with either human-specific or chimpanzee-specific enhancers. Interestingly, functional validation of enhancer activity suggested that enhancer divergence is largely a result of *cis*-sequence changes, rather than differences in the *trans*-regulatory landscape (comprising transcription factors, cofactors and regulatory RNA repertoire) between species. This study denotes how morphogens and other molecules conserved across vertebrates can be differentially regulated on the enhancer level to build species-specific features and provides solid support for *cis*-regulatory evolution being an essential mechanism of morphological evolution and speciation.

Exquisite facial variability is found in humans, where phenotypic diversity evolved together with specialized cognitive functions and represents a powerful tool for mutual recognition, social interactions and communication [8,100]. The desire to understand the genetic architecture behind human face formation, led White and colleagues to perform multivariate genome-wide association analysis of over 8000 individuals. It is important to highlight that while muscles and adipose tissues contribute to facial appearance, facial shape is largely defined by the geometry of the underlying skeletal elements. Thus, the observations from this study explore how genetic variants influence skull shape. The authors associated 203 genome-wide signals to normal-range facial variation. Identified regions were enriched for enhancer activity in the neural crest cells and for genes relevant for craniofacial and limb development. Head and limb formation share conserved developmental and molecular mechanisms (reviewed by Schneider *et al.* [101]); thus, it is not surprising that the regions identified in this study are relevant for pathways employed in both morphogenetic processes (e.g. WNT, Hedgehog or TGFβ). Several variants were found to affect the same facial segment, indicating that multiple enhancers act jointly on the formation of a specific trait.

A recent study recognized the need to understand the regulatory landscape in relevant tissues and specific developmental stages. In humans, the majority of craniofacial development occurs in the first 10 weeks [18]. Wilderman *et al.* [102] profiled multiple chromatin marks in primary human facial tissues across several developmental stages from 4.5 to 8 weeks post conception. The authors first identified chromatin states (activated or poised) of genes involved in craniofacial development. Large active chromatin regions also covering promoters were previously linked to critical developmental regulators [51]. Subsequently, the authors reported enrichment for craniofacial enhancers and super enhancers that were enriched in regions coding for known developmental genes (members of the *WNT*, *FZD*, *ALX*, *DLX*, *TBX* families). This suggests that the identified regulatory regions are likely controlling patterning of bones in the forming face. The authors took advantage of previous studies that reported association of regulatory elements with certain facial shapes and found strong enrichment in later developmental tissues, which highlights the existence of specific regulatory events related to facial shape acquisition, while enhancers of typically patterning genes were mostly found in early developmental stages. This excellent study shows the need for primary tissue-specific and stage-specific analysis of chromatin states and regulatory landscapes.

Fossil findings can also fuel our understanding of the genetic changes behind phenotypic evolution, although exact information on the gene expression and epigenomic modifications is missing. The discovery of the genomes of human ancestors—the Neanderthal and Denisovan genomes—was used to identify regulatory elements that differ between archaic and modern humans [103]. The study revealed that the majority of genetic variation between modern and archaic humans is found in non-coding sequences [103], thus providing evidence that variation is preferentially linked to elements with potential regulatory function and that the evolution of *cis*-regulation underlies the phenotypic evolution of modern humans. Employing a massively parallel reporter assay in embryonic stem cells, neural progenitors and osteoblasts, the activity of modern human-specific non-coding (single-nucleotide) variants was tested [104]. These three cell types were selected based on information we can deduct from fossil findings, namely skeletal differences, and their correlation with the basic nervous system architecture (such as size and position). From the active variants that exhibited regulatory activity, 23% of sequences controlled differential expression between the groups. The sequences of modern human variants that were found to upregulate gene expression were prominently associated with genes related to vocal apparatus, craniofacial features, bone development, brain development and learning ability.

Evolution of novel morphology requires changes in gene expression patterns that are translated into developmental processes. The appearance of morphological innovation does not necessarily rely upon an acquisition of novel genes and instead the emergence of novel regulatory elements is reportedly sufficient to drive the origin of such novelty [21,105]. One such example and a hallmark of mammalian evolution is the neocortex [106]. This six-layered thick brain compartment mediates complex cognitive and motor tasks. Since the early mammals, the proportion of the neocortex extended, and the human neocortex represents 80% of brain volume [107]. Emera *et al.* [108] aimed to address the role of regulatory sequences in neocortex evolution. After identification of enhancers active in neocortical development in mouse and human, the authors found that 20% of these elements emerged in the stem mammalian lineage and are enriched in regions coding for genes associated with cell migration, axon guidance and cell signalling (especially ephrin and sematophorin). These processes and signalling are linked to the origin and function of the neocortex, and thus this study supports the idea that mammal-specific evolution of regulatory functions contributed to the emergence and substantial diversification of the neocortex in mammals. For this review, it is important to mention that nervous system morphogenesis is tightly linked to skull formation and morphology (reviewed by Adameyko & Fried [109], Richtsmeier & Flaherty [110] and Marcucio *et al.* [111]). Multiple developmental organizers or so-called ‘signalling centres’ are hosted within the developing nervous system and orchestrate the coordinated growth and integration of these two essential structures. Recent investigation into the effect of the nervous system-derived morphogen Sonic Hedgehog on mouse skull formation highlighted the importance of its enhancer-driven spatio-temporal distribution and expression level for the establishment of morphology [88]. It is thus evident that *cis*-regulatory evolution within the nervous system prominently impacts skull morphological evolution.

## 6. Current challenges and future research directions

Craniofacial shape is highly polygenic, with heritability up to 70% in humans [11,112]. Until recently, most work identified individual genes involved in craniofacial development. While this approach provides valuable information about individual genes and their function in particular developmental events, it does not significantly contribute to the understanding of complex programmes forming high-dimensional phenotypes. The intertwining of genes and respective signalling pathways with an intricate *cis*-regulatory landscape is key for orchestrated morphogenetic processes.

While comparative studies of mammalian genomes shed light on the structural variance of known regulatory elements and gave us a hint as to the extent of variation, to fully understand the genome-wide atlas of active enhancers, there is a need to perform systematic studies covering corresponding developmental stages along an extended developmental timeline and across species. Current progress is hindered by many obstacles, such as identification of matching stages across numerous mammalian species with significant developmental heterochrony, and the feasibility of obtaining such samples. In addition, the conventional approach to functional validation of candidate enhancers has its limits. Validation relies on introduction of the enhancer, a minimal promoter and a reporter cassette into a model organism that is amenable to modifications, such as the mouse. However, the insertion of an enhancer sequence from a phylogenetically distant species without its natural DNA organization into a species with a substantially different *trans*-regulator landscape does not provide reliable information as to whether the observed change of gene expression is due to the evolution of the enhancer, the presence of distinct *trans*-regulators or the effect of higher-order DNA structure (such as topologically activating domains) [105].

In addition, due to heterotopy and heterochrony occurring between species, there is intrinsically hidden heterogeneity of cell types and tissues at a given developmental stage. Changes in cell fate specification and cell behaviour such as migration and oriented cell division will initiate a sequence of further alterations and modify morphogenesis on various levels. Therefore, single-cell resolution would provide unprecedented insight into the developmental regulatory atlas, and allow enhancer activity to be observed in each cell (and cell type), together with the composition of stage- and cell-type-specific array of transcription factors.

Combining advanced chromosome conformation capture and epigenetic modification assays enables genome-wide mapping of enhancer activation along development [113]. However, functional validation of enhancer function is required to understand the effect of individual regulatory elements on phenotypic trait formation. A thoughtfully selected paradigm (e.g. a single morphogen that evinces species-specific 4D expression pattern and is controlled by several enhancers) would enable the understanding of the modularity of enhancer action and its impact on trait formation. A series of functional validations would be required to understand the effect of individual sequences in each of the developmental steps. This would need to be complemented with careful analysis of their impact on cell fate specification, proliferation, polarity and how such effects project onto neighbouring cells and tissues, and timing of morphogenetic processes.

Comparing mammalian species with significant developmental heterochrony might provide insight into the effect of *cis*-regulatory evolution on different birth timing and feeding modes that are reflected in the craniofacial morphology of newborns. For instance, marsupials possess a highly functional craniofacial region at the time of their birth, which is comparable to the mouse embryonic developmental stage of 10–12 days. Extreme marsupial prematurity at the time of birth is compensated by a series of morphological and functional adaptations enabling development to continue in the mother′s pouch. Systems allowing locomotion, breathing and feeding, together with sensory capacity, show strikingly accelerated development in marsupials compared to eutherian mammals. Interestingly, the differences between marsupials and eutherian mammals have not arisen based on de novo gene evolution but rather are the result of insertions and repurposing of TEs and retroviruses [114–117]. These genomic structural changes driven by TEs and retroviruses facilitated changes in existing GRNs, mostly within non-coding sequences, which led to the emergence of novel species-specific gene expression patterns, new morphologies and functional adaptations of the skull [118]. Therefore, comparative and functional genomics of marsupial and placental development represents a strategy to uncover the evolutionary origin and consequences behind distinct reproductive strategies, adaptations and speciation in mammals.

Until today, direct comparisons of corresponding stages and tissues have not yet been systematically performed across mammals. There is a need to explore tissue- or cell-type-specific *cis*-regulatory landscapes and their evolution in a developmentally relevant stage and tissue, and to compare such data across several species with distinct facial morphology. Such investigations are likely to bring us profound understanding of enhancer evolution and its impact on morphological variance in complex structures. Understanding the precise control of gene expression in embryonic time and space with a focus on genes that build and pattern body plans may provide a holistic picture of gene regulation in a dynamic morphogenetic process.

## 7. Conclusion

The mammalian skull is an excellent model to study morphological evolution, especially in the context of divergence of *cis*-regulatory sequences. Mammals possess exceptional craniofacial variability that is linked to the acquisition of specific functions and adaptations to diverse environments. Simultaneously, mammalian craniofacial development is well studied compared to other vertebrates, and there is substantial conservation of both genes and developmental processes. The current goal is to grasp how conserved developmental programmes can be calibrated to generate a spectrum of shapes and how regulatory landscapes tune spatio-temporal gene expression and translate into morphogenetic processes. Comparative investigation of morphological integration of tissues and organs in forming the mammalian head identifies the points of divergence of developmental processes between species. A combination of thoughtfully designed paradigms, functional genomics, morphometrics and functional validations will allow us to reveal mechanisms underlying this divergence and the evolution of the skull in mammals.

Understanding the evolution of *cis*-regulation and its effects on craniofacial morphogenesis is essential for the additional aspects. A number of neurological and neurodevelopmental syndromes manifest by facial abnormalities, and a large proportion of these disorders are not linked to a mutation in coding regions. With better knowledge of the relationship between enhancer, gene expression profiles and phenotypes we may be able to predict and improve the management of human congenital craniofacial disorders. At the same time, modelling approaches might be used to predict facial shape and revolutionize the forensic sciences, possibly allowing deduction of facial appearance from genetic information.

## Data Availability

This article has no additional data.
